# Treatment beyond four cycles of first line Platinum and Etoposide chemotherapy in real-life patients with stage IV Small Cell Lung Cancer: a retrospective study of the Merseyside and Cheshire Cancer network

**DOI:** 10.1186/s12890-019-0948-x

**Published:** 2019-11-01

**Authors:** Mostafa Sallam, Helen Wong, Carles Escriu

**Affiliations:** 1 The Clatterbridge Cancer Centre, Clatterbridge Road, Bebington, Wirral, CH63 4JY UK; 2 University of Liverpool, L69 3BX, Liverpool, UK

**Keywords:** Lung neoplasm, Small cell lung carcinoma, Drug therapy, Observational study, Antineoplastic combined chemotherapy protocols

## Abstract

**Background:**

Dose intensity and dose density of first line Platinum and Etoposide (PE) do not influence Overall Survival (OS) of Small Cell Lung Cancer (SCLC) patients. The effect of treatment length, however, remains unclear. Current guidelines recommend treating beyond 4 cycles -up to 6-, in patients that respond to and tolerate systemic treatment. This has led to variable practice both in clinical practice and clinical research. Here we aimed at quantifying the possible clinical benefit of the extended regimen in our real-life patients treated with PE doublet.

**Methods:**

Of all patients with SCLC treated in our network with non-concurrent first line PE chemotherapy between 2008 and 2015, we identified and described patients that received 4 cycles (4c) or more (> 4c), and analysed patients with stage IV disease.

**Results:**

Two hundred forty-one patients with stage IV had 4c and 69 had > 4c. The latter were more likely to have sequential thoracic radiotherapy, which suggested a lower metastatic burden. Nevertheless, there were no statistically significant differences when comparing clinical outcomes. The median Duration of Response (DoR; time from last chemotherapy cycle to progression) was 5 months in both groups (HR 1.22; 95% CI 0.93–1.61). Median Progression Free Survival (PFS; time from diagnosis to radiological progression) was 8 months (4c) versus 9 months (> 4c) (HR 0.86; 95% CI 0.66–1.13) and median OS was 11 versus 12 months (HR 0.86, 95% CI 0.66–1.14).

**Conclusion:**

Our results highlight a lack of clinical benefit by extending first line PE treatment in stage IV disease, and support limiting treatment to 4 cycles until superiority of a longer regimen is identified in a randomised study.

## Background

Small Cell Lung Cancer (SCLC) is the most common neuroendocrine tumour of the lung [1] and accounts for 10% of all lung cancers [2]. Its incidence is associated with smoking, almost two thirds of patients present with advanced disease [3], and although response rates to chemotherapy are high, the benefit is short-lived. With platinum and etoposide (PE) chemotherapy combination in extensive disease the median progression-free survival (PFS) is only 5.5 months and the median overall survival (OS) under 10 months [4]. These figures underline the need to optimise oncological treatment in SCLC, so that survival benefit is maximised whilst unnecessary treatment and toxicity are avoided.

When SCLC is not amenable to concurrent chemoradiotherapy, international guidelines advice 4 to 6 cycles of first line systemic Platinum (Cisplatin or Carboplatin) and Etoposide combination (PE) [5, 6]. Prophylactic Cranial Irradiation (PCI) [7], and sequential thoracic radiotherapy may be considered in patients with either limited [8, 9] or extensive disease [10].

So far, in SCLC more chemotherapy does not translate in more clinical benefit. Strategies increasing chemotherapy dose intensity, dose density, peak dose or total dose regimens have been studied extensively and failed to provide a survival advantage [11]. Randomised clinical trials differ in identifying a survival benefit with maintenance Topotecan or Etoposide after 4 cycles of platinum combination [12, 13]. Two meta-analysis [14, 15] concur with a potential impact in PFS in extensive disease but differ in its survival benefit. Although maintenance treatment may be common practice in some countries [14] international guidelines discourage its use [5, 6].

The length of first line PE treatment, however, remains controversial. In the early 80’s, 6 cycles of Vincristine, Doxorubicin and Cyclophosphamide (CAV) were the standard of care. A decade later Roth et al. [16] run a randomized phase III study with over 437 eligible patients. They compared 4 cycles of Cisplatin and Etoposide with 6 cycles of CAV and 6 cycles of a hybrid regimen of CAV and Cisplatinum-Etoposide. No PFS or survival differences were identified, but a higher rate of infection was found in CAV containing regimens. The only study that compared 4 versus 6 cycles of Cisplatin and Etoposide chemotherapy was published a few years later by Veslemes et al. [17]. Sixty-nine of the 70 randomised patients were analysded, and only 46 had extensive disease. The primary objective of the study was undisclosed, and there was no pre-planned subgroup analysis. Anyhow, no significant survival differences were identified other than a trend to survival gain in patients with extensive disease at the expense of higher toxicity. Chasing this possible benefit with a longer regimen appeared less intricate when outpatient Carboplatin containing doublet administration showed similar clinical benefit than the then inpatient Cisplatin based treatment [18, 19].

International guidelines [5, 6] continue to encourage the use of a range of chemotherapy cycle numbers. Nonetheless, this promotes variability in real-life practice and clinical research. For example, some randomised studies may use 4 cycles as standard of care control [12, 13], while others may design their study to optimise the delivery of 6 cycles [20].

Perhaps due to the limited evidence available, the optimal length of first line platinum combination treatment in recent reviews is either omitted [21–23] or maintenance treatment is discussed instead [24]. The drastically inferior benefit of second line options [5, 24] and the low impact of novel biological approaches so far [23] are factors that may influence treatment attitudes towards the length of this first line setting. This is unlikely to change in spite of the recent introduction of first-line immune therapy combination with 4 cycles of chemotherapy [25]: as long as the backbone of cytotoxic chemotherapy remains, the possibility of extending cytotoxic chemotherapy to 6 cycles will remain unchanged for as long as the perception of benefit remains unchallenged. Furthermore, extending PE chemotherapy in patients that are not amenable to first-line immune therapy combination may be the only option to attempt to optimise their survival benefit. In this context a prospective study is unlikely to materialize, and retrospective analysis of real-life patients is the only method available to identify the optimal treatment length.

The Merseyside and Cheshire Cancer Network serves a population of 2.3 million with non-surgical oncology provision and receives over 1000 new lung cancer referrals per year. Of those that receive treatment in our centre, audit data is recorded prospectively. Practice amongst oncologists is diverse and some advocate for treatment continuation beyond 4 cycles in selected patients. Here we aim at characterising that subpopulation and quantifying the possible survival gain over a four-cycle regimen.

## Methods

This was a retrospective observational study of the Merseyside and Cheshire Cancer Network. Audit approval was obtained ahead of data collection according to local policy. Patients referred with pathologically confirmed small cell lung cancer and treated with Platinum and Etoposide combination regimens over an 8-year period (2008–2015) were identified. The first date was chosen to optimise prospective data record consistency, and the latter to allow mature one-year survival data. Two thousand fifteen also preceded the use of standard consolidation radiotherapy in extensive disease [10]. Patients treated with concurrent chemo-radiotherapy, non-platinum-based combination or single agent platinum, were excluded.

Epidemiological, pathological, treatment and survival data were extracted from the prospective database and confirmed retrospectively. Radiological response and progression was collected retrospectively. Lung radiotherapy was defined as radiotherapy administered to the primary lung tumour any time after systemic chemotherapy regardless of intent. Analysis on treatment outcomes were performed for stage IV SCLC (*n* = 69 for > 4c and *n* = 241 for 4c). Progression was defined as the date of radiological progression. Duration of Response (DoR) was defined as the time from the date of administration of the last cycle of chemotherapy to the date of progression or death. Progression Free Survival (PFS) was defined as the time from diagnosis to progression or death. Overall Survival (OS) was defined as time from diagnosis to death or last patient review.

Chi-square contingency analysis was performed to identify epidemiological or treatment variables. PFS and OS curves were constructed using the Kaplan-Meier method, and compared for significance using the log-rank test. A cox proportional hazards model was used to generate univariate hazard ratios (HR) and 95% confidence intervals. A two-sided *p* < 0.05 was considered as statistically significant. All statistical analyses were carried out using the statistics software IBM SPSS v.24.0 (SPSS Inc.).

This paper was written according to the STROBE criteria version 4.

## Results

### Patient characteristics

**Fig. 1:**
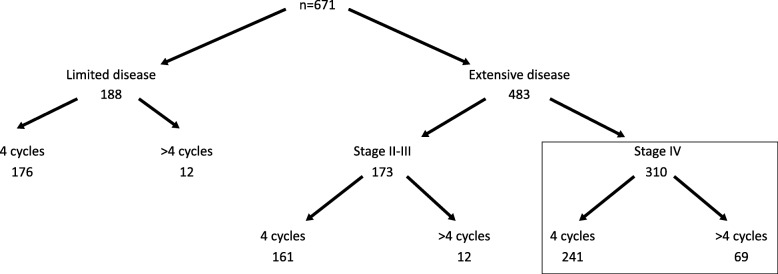
Patient flow diagram

Of the 671 patients that received systemic PE combination treatment without concurrent radiotherapy, 93 were selected to have more a longer regimen, of which 24 (26%) had an initial stage of I-III -half of them (*n* = 12, 13%) with limited disease-, and 69 (74%) had stage IV disease. When comparing patients that had 4 cycles (4c) (*n* = 578) versus those that went on to have more than 4 (> 4c) (*n* = 93), significant differences were identified in their co-morbidity, stage and disease extent (see Table 1) as well as the proportion of second line chemotherapy received (see Table 2), but there were no differences in their best response rates to treatment. Patients selected to have a longer treatment had less co-morbidity, more advanced stage and were eventually less likely to receive second line chemotherapy. These differences were not apparent when comparing patients that presented with stage IV disease, of which 241 completed 4 cycles and 69 completed more than 4. Hence, we decided to focus in these more homogeneous sub-population of patients with stage IV disease for outcome analysis (see Fig. 1).

**Table 1:** Demographic characteristics. Percentage relative to the total at the top row is presented in brackets. Chi-square contingency analysis was performed where indicated. Statistically significant results are highlighted in italic and an asterisk

	All patients			Extensive Disease			Stage IV		
	>4 cycles	4 cycles	*p* value	>4 cycles	4 cycles	*p* value	>4 cycles	4 cycles	*p* value
Total N	93	578		81	402		69	241	
Sex, n (%)			0.115			0.113			0.218
Male	48 (51.6)	246 (42.6)		43 (53.1)	174 (43.3)		37 (53.6)	107 (44.4)	
Female	45 (48.4)	332 (57.4)		38 (46.9)	228 (56.7)		32 (46.4)	134 (55.6)	
Age, Avg [Min-Max]	65 [39–81]	66 [37–88]	0.847	65 [39–80]	66 [37–88]	0.358	65 [39–80]	65 [41–87]	0.812
PS, n (%)			0.426			0.367			0.192
PS0	14 (15.1)	64 (11.1)		14 (17.3)	45 (11.2)		14 (20.3)	29 (12)	
PS1	41 (44.1)	288 (49.8)		33 (40.7)	189 (47)		26 (37.7)	122 (50.6)	
PS2	34 (36.6)	205 (35.5)		30 (37)	149 (37.1)		25 (36.2)	83 (34.4)	
PS3	3 (3.2)	20 (3.5)		3 (3.7)	18 (4.5)		3 (4.3)	6 (2.5)	
PS4	1 (1.1%)	1 (0.2%)		1 (1.2%)	1 (0.2%)		1 (1.4%)	1 (0.4%)	
Comorbidities, n (%)			0.01*			0.017*			0.140
None	26 (28)	85 (14.7)		21 (25.9)	60 (14.9)		17 (24.6)	37 (15.4)	
Mild	40 (43)	320 (55.4)		35 (43.2)	222 (55.2)		33 (47.8)	134 (55.6)	
Moderate	24 (25.8)	162 (28)		23 (28.4)	111 (27.6)		17 (24.6)	66 (27.4)	
Severe	3 (3.3)	11 (1.9)		2 (2.4)	9 (2.2)		2 (2.8)	4 (1.7)	
Stage, n (%)			< 0.001*			< 0.001*			
Stage I	1 (1.1)	11 (1.9)		–	–		–	–	–
Stage II-III	23 (24.7)	326 (56.4)		12 (14.8)	161 (40)		–	–	
Stage IV	69 (74.2)	241 (41.7)		69 (85.2)	241 (60)		69 (100)	241 (100)	
Disease extent, n (%)			< 0.001*						
Limited	12 (12.9)	176 (30.4)		–	–		–	–	
Extensive	81 (87.1)	402 (69.6)		81 (100)	402 (100)		69 (100)	241 (100)	
SVCO at presentation	4 (4.3)	36 (6.2)	0.466	4 (4.9)	30 (7.5)	0.633	2 (2.9)	14 (5.8)	0.538

**Table 2:** Treatment characteristics. Percentage relative to the total at the top row are presented in brackets. Chi-square contingency analysis was performed where indicated. Statistically significant results are highlighted in italic and an asterisk

	All patients			Extensive Disease			Stage IV		
	>4 cycles	4 cycles	*p* value	>4 cycles	4 cycles	*p* value	>4 cycles	4 cycles	*p* value
Total N	93	578		81	402		69	241	
1st Line Chemotherapy, n (%)			0.148			0.557			0.689
Cisplatin Etoposide	2 (2.2)	37 (6.4)		2 (2.5)	20 (5)		1 (1.4)	8 (3.3)	
Carboplatin Etoposide	91 (97.8)	541 (93.6)		79 (97.5)	382 (95)		68 (98.6)	233 (96.7)	
Cycle Number, n (%)									
4 cycles	–	578 (100)		–	402 (100)		–	241 (100)	
5 cycles	13 (14)	–		13 (16)	–		11 (15.9)	–	
6 cycles	80 (86)	–		68 (84)	–		58 (84.1)	–	
Average Cycles	5.9	4		5.8	4		5.8	4	
Response, n (%)			0.461			0.9807			0.538
Progression	3 (3.2)	13 (2.2)		3 (3.7)	12 (3)		2 (2.9)	8 (3.3)	
Stable Disease	12 (12.9)	53 (9.2)		11 (13.6)	37 (9.2)		10 (14.5)	25 (10.4)	
Partial Response	57 (61.3)	412 (71.3)		51 (63)	288 (71.6)		44 (63.8)	74 (72.2)	
Complete Response	21 (22.6)	100 (17.3)		16 (18.9)	62 (15.4)		13 (18.8)	34 (14.1)	
Radiotherapy to lung, n (%)	43 (46.2)	254 (43.9)	0.736	35 (43.2)	146 (36.3)	0.259	27 (39.1)	61 (25.3)	0.033*
PCI, n (%)	37 (39.8)	240 (41.5)	0.821	29 (35.8)	156 (38.8)	0.707	24 (34.8)	96 (39.8)	0.486
2nd line Chemotherapy, n (%)	17 (18.3)	167 (28.9)	0.034*	17 (21)	108 (26.9)	0.271	16 (23.2)	62 (25.7)	0.754
CarboEtop	7 (7.5)	88 (15.2)	0.804	7 (8.6)	47 (11.7)	0.796	6 (8.7)	29 (12)	1.000
Topotecan	4 (4.3)	15 (2.6)		4 (4.9)	5 (1.2)		4 (5.8)	4 (1.7)	
CAV	6 (6.5)	62 (10.7)		6 (7.4)	42 (10.4)		6 (8.7)	28 (11.6)	
3rd line Chemotherapy, n (%)	4 (4.3)	26 (4.5)		4 (4.9)	18 (4.5)		4 (5.8)	9 (3.7)

In patients with stage IV both treatment groups had a similar sex distribution and an average age of 65; around 40% had PS2 or more, and almost 30% had moderate or severe co-morbidities. Most patients received carboplatin-based chemotherapy: 97% in the 4c group and 99% in the > 4c. In that latter group, 84% completed 6 cycles, reaching an average of 5.8 cycles. Again, response rates were similar in both groups.

Whereas PCI rates were less than 40% in both groups, the rates of radiotherapy to the lung were higher in the patients treated with a longer regimen (25% in 4c versus 39% in > 4c). The intent of thoracic radiotherapy was in all cases palliative*.* There were no differences in second line treatment rates, and the proportion of platinum re-challenge was 12% in the 4c group versus 9% in the > 4c.

### Treatment outcomes

The similar rate of platinum re-challenge was concordant with the clinical outcomes measured: there were no significant differences in duration of response (DoR), progression free survival (PFS) or overall survival (OS) in patients with stage IV disease.

**Fig. 2:**
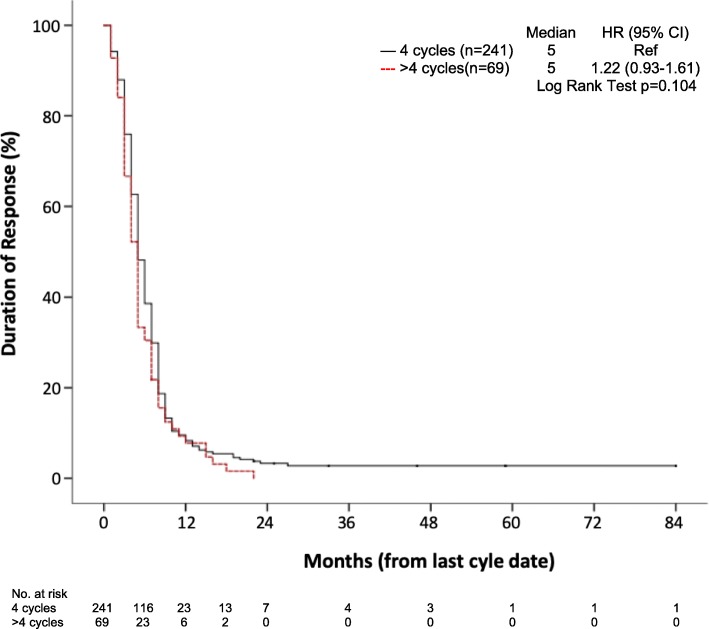
Kaplan Meyer curve of time from last chemotherapy cycle to date of progression or death (DoR; Duration of Response)

DoR curves (time from the last cycle administration to disease recurrence) for patients that completed first line PE treatment are shown in Fig. 2, with a median DoR of 5 months in both groups. The HR for > 4c was 1.22 (95% CI 0.93–1.61; *p* = 0.104), and the 6 month DoR was 48% for the 4c group versus 33% for the > 4c.

**Fig. 3:**
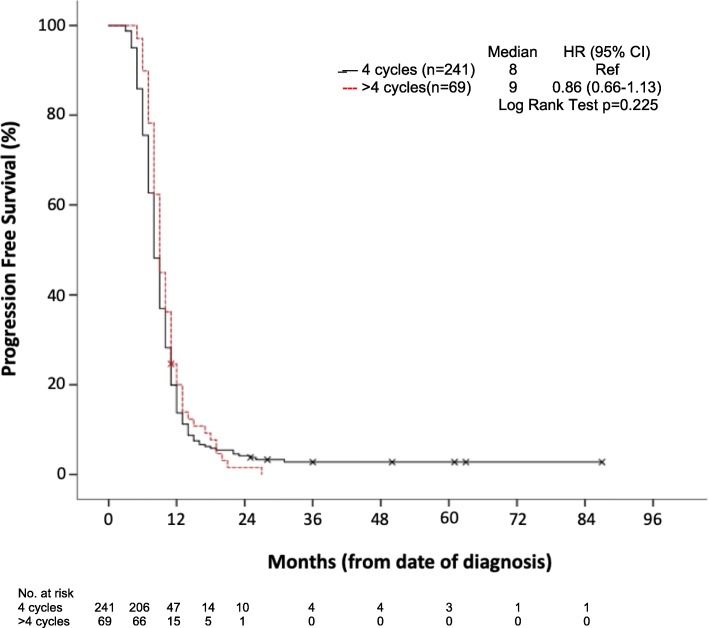
Kaplan Meyer curve of time from diagnosis to date of disease progression or death (PFS; Progression Free Survival)

PFS curves (see Fig. 3) show a median PFS of 8 (4c) versus 9 months (>4c), with an HR for >4c of 0.86 (95% CI 0.66–1.13; *p* = 0.225). Respectively, the 6-month PFS was 86% versus 96%, and the 1 year PFS 20% versus 23%.

**Fig. 4:**
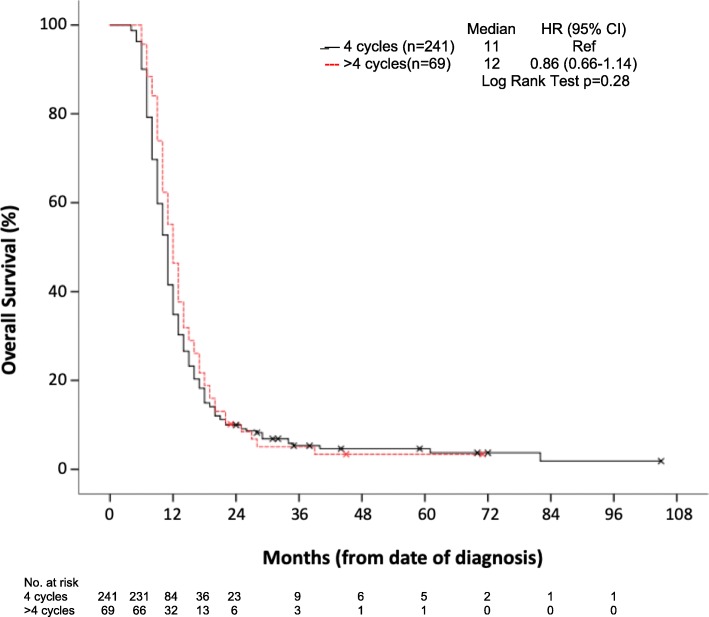
Kaplan Meyer curve of time from diagnosis to death (OS; Overall Survival)

The median OS (see Fig. 4) was 11 months for the patients in the shorter treatment and 12 months for the longer schedule, with an HR for >4c of 0.86 (95% CI 0.66–1.14; *p* = 0.28). The one-year OS was 35 and 46% respectively.

## Discussion

To our knowledge, this is the largest study analysing the effect of extending platinum doublet treatment beyond 4 cycles in patients with stage IV SCLC disease. Our selected real-life patients with stage IV SCLC did not improve disease control or survival by extending first line platinum and etoposide combination treatment beyond 4 cycles.

The retrospective nature of the study was an important limitation, restricting accurate quantification of the timing of the treatment length decision - at presentation or after 3–4 cycles -, and treatment toleration. The latter was related to inter-consultant differences in patient assessment as well as changes in standard toxicity chemotherapy nurse documentation during the study time. Noticeably, there was a low proportion of patients that went on to complete more than 4 cycles of chemotherapy: only 93 (14%) of the 671 initial population and 69 (22%) of the 310 patients with stage IV. These numbers suggested high patient selection, a bias that would favour the longer treatment group. Nevertheless, radiological response (the one measurable selection criteria available) was similar in patients with stage IV regardless of treatment length. This may have been due to (a) inaccurate patient selection on radiological assessment of response, (b) a fundamental reliance on treatment toleration, or (c) a decision of treatment length intent at presentation based on non-recorded criteria such as clinical relevance of metastatic burden.

The latter possibility may have been evident when exploring differences in subsequent treatment between the groups. 39% (*n* = 27) of patients in the >4c group went on to have palliative radiation to the lung, whereas only 25% (*n* = 61) of the 4c group received the same treatment. The clinical benefit in extensive disease is limited [10], but may suggest a lower clinical relevance of the metastatic burden in this particular subpopulation. In spite of this bias that would favour the >4c population no differences in clinical outcomes were observed.

Another potential bias inherent to retrospective studies is a suspected variability in the timing of patient review and investigations during follow up, influencing the diagnosis of recurrence. Still, there is no reason to expect differences in follow up related to the length of the first line treatment, hence we assumed equal distribution of this bias in both treatment groups.

As expected, our PFS and OS values were better than those previously reported [4]. Patients that received 3 or less treatment cycles were not included and therefore their outcome did not burden the clinical measures of the populations studied here.

Caution is required when extrapolating our results to patients with early stage disease, as there were only small numbers in our long treatment cohort, and they were excluded from clinical outcome calculations (see Fig. 1). However, in view of the observed lack of benefit of a longer treatment in extensive disease, and the lack of prospective evidence of benefit in limited stage [17], it would be reasonable to question the benefit of treating beyond 4 cycles in patients with limited disease undergoing sequential chemoradiotherapy.

## Conclusion

Our real-life data in stage IV disease does not support treatment beyond 4 cycles of PE combination. That is consistent with the lack of benefit seen in randomised prospective studies comparing 4 cycles with 6 cycles of either a hybrid regimen [16] or cisplatin and etoposide [17]. Limiting treatment to 4 cycles may contribute to avoid unnecessary hospital visits and toxicity, optimise treatment cost-effectiveness and perhaps increase the focus on recruitment into clinical trials exploring novel strategies [26] with the potential of improving survival outcomes.

## Data Availability

The datasets used and analysed during the current study are available from the corresponding author on reasonable request.

## Abbreviations

> 4c: More than four cycles
4c: Four cycles
CAV: Vincristine, Doxorubicin and Cyclophosphamide
CI: Confidence Interval
DoR: Duration of Response
HR: Hazard Ratio
OS: Overall Survival
PCI: Prophylactic Cranial Irradiation
PE: Platinum and Etoposide
PFS: Progression-Free Survival
SCLC: Small Cell Lung Cancer
